# From Lifestyle to Social Determinants: New Directions for Community Health Promotion Research and Practice

**Published:** 2007-06-15

**Authors:** Nicholas Freudenberg

**Affiliations:** Hunter College, City University of New York

Health promotion provides a powerful tool for improving health in the 21st century, but researchers and practitioners have yet to achieve consensus on its scope. Globalization, urbanization, an aging population, and rising rates of chronic diseases are creating new health challenges throughout the world. How can health professionals respond to these changing circumstances? What are the relevant paradigms for promoting health today? How can universities help move health promotion into a new era?

In the last 50 years, the dominant view in the United States has been that lifestyle is the major remediable cause of ill health (1). Researchers view lifestyle as a series of choices that individuals make about food, exercise, substance use, and sexual activity. Practitioners create individual, community, and media interventions to persuade people to change these behaviors to reduce their risk for disease. While most observers acknowledge that social forces influence these choices, most interventions focus on changing individuals.

At the March 2006 meeting of the National Expert Panel on Community Health Promotion (2), participants articulated the limitations of this approach. The approach fails to analyze the determinants of lifestyle, thus missing opportunities for more "upstream" interventions (3). It blames individuals at highest risk for ill health, even when their choices have been constrained by public policies and corporate practices. Also, this approach is inefficient, requiring health promoters, like Sisyphus, to push every person engaged in unhealthy behavior up the steep hill of disease-promoting environments toward health at the top, rather than leveling the incline by changing policy.

Helping individuals to change unhealthy behavior will always be part of health promotion. But if the United States is to achieve health goals such as reducing the burden of chronic disease, eliminating health disparities, and engaging more constituencies in promoting health, it needs to reconsider its approaches to health promotion.

Universities can help to forge more effective approaches by taking on four tasks. First, academics can help reframe our view of lifestyle. Individuals make choices in a social context. Rather than regarding lifestyle as the prime cause of health problems, we need to analyze the determinants of lifestyle. For example, the advertising, pricing, and retail practices of the food, alcohol, and tobacco industries profoundly influence health choices (4). Public policies on recreation, transportation, and urban development shape opportunities for physical activity (5). Research on the causes of lifestyle choices will help to open new avenues for health promotion.

The second task is to analyze the social processes that create poor health in order to identify new intervention opportunities. For example, epidemiologic evidence demonstrates that more education is associated with better lifetime health, yet in many American cities half the young people who enter high school fail to graduate on time and many never finish high school (6). Improving school completion rates could improve population health, especially among the most disadvantaged, and reduce disparities in health. Yet rarely do health agencies make reducing school dropout a priority. Moreover, evidence suggests that pregnancy prevention programs, comprehensive health and sexuality education, school-based clinics, mental health services, and violence reduction programs can improve adolescent health and reduce school dropout by engaging young people with school, connecting them to caring adults, reducing their absenteeism, and increasing their feelings of safety, which are all associated with reduced dropout (7). By developing new alliances with educators and making school completion a goal of health promotion, health professionals can improve population health and social well-being.

The third task is to engage more constituencies in health promotion. Not only can schools, health departments, health providers, faith groups, and community organizations join in promoting health but so can employers, labor unions, elected officials, universities, social movements, immigrant organizations, and disenfranchised groups. By framing health as an economic, environmental, social justice, and moral issue, health professionals can enlist more stakeholders in the process. Academics can contribute to this goal by studying the process of health mobilization and identifying the characteristics of effective strategies.

Finally, the development of effective health promotion will require health professionals with new skills. These skills include an ability to analyze health problems at various levels of social organization, reframe health problems to engage diverse constituencies in the health promotion process, advocate for policy change in the face of opposition by special interests, and evaluate the success of health promotion interventions that seek changes in fundamental determinants.

Academic public health programs can help to forge a 21st-century practice and research agenda for health promotion by recruiting students from more diverse communities; strengthening training in social analysis and policy advocacy; and developing partnerships with communities, policy makers, and advocates.
